# Supplementary material for: Altered microRNA Transcriptome in Cultured Human Liver Cells upon Infection with Ebola Virus
Source: Int J Mol Sci. 2021 Apr 6;22(7):3792. doi: 10.3390/ijms22073792 (PMC8038836; doi:10.3390/ijms22073792)
Supplement: Supplementary file 1 [file ijms-22-03792-s001.zip › Supplementary_File/C_ GO_Analysis_Results/16-30nt_go_Mayinga-96h-Huh7_vs_Control-96h-Huh7_down.mature_mirna_targets/CC_result(Human).html]

| GO.ID | Term | Ontology | Count | Pop.Hits | List.Total | Pop.Total | Fold.Enrichment | Pvalue | FDR | Enrichment.Score | Gene.Ratio | GENES |
| --- | --- | --- | --- | --- | --- | --- | --- | --- | --- | --- | --- | --- |
| GO:0045202 | synapse | Cellular component | 38 | 880 | 395 | 18698 | 2.04408515535098 | 2.49045398559216e-05 | 0.0213929997362367 | 4.60372147801834 | 0.0962025316455696 | TBC1D24//FGFR2//NLGN1//LRRTM1//BSN//ACTR2//CHRM1//FBXO45//DBNL//GRIN2B//PALM//RTN4//BCR//LRP8//SYNPR//SYPL2//ATP6V1G2//SYT9//ABCC8//CPLX1//AP1S1//CPLX3//CANX//ZNF804A//ELK1//KCNIP3//KCNA6//GABRR1//GRIA3//SSPN//FZD3//P2RX2//GRIPAP1//LYPD6//HTR3E//ITGA5//CNTN2//NDFIP1// |
| GO:0000139 | Golgi membrane | Cellular component | 32 | 729 | 395 | 18698 | 2.07788022434061 | 8.1872373739687e-05 | 0.0309213818619268 | 4.08686261785396 | 0.0810126582278481 | TMED2//NUCB1//ST3GAL3//AP1S1//ARFIP2//HLA-DPA1//VPS53//CALN1//SYS1//STEAP2//YKT6//KDELR1//VPS45//GBP4//DBNL//ST8SIA5//GOLGA7B//NCAM1//NDFIP2//GALNT10//SLC30A6//RAB2A//XYLT2//SLC35A2//TNKS2//NDFIP1//FAM57B//CREB3L3//NDST2//NDST3//HS6ST1//GOSR2// |
| GO:0044456 | synapse part | Cellular component | 32 | 740 | 395 | 18698 | 2.04699281560041 | 0.000107990856328033 | 0.0309213818619268 | 3.96661301501667 | 0.0810126582278481 | SYT9//ABCC8//FZD3//BSN//NLGN1//CPLX3//ZNF804A//P2RX2//PALM//FBXO45//CHRM1//GABRR1//GRIA3//GRIN2B//LRRTM1//BCR//SSPN//SYNPR//SYPL2//ATP6V1G2//CPLX1//AP1S1//TBC1D24//ELK1//KCNIP3//KCNA6//CANX//GRIPAP1//ACTR2//DBNL//RTN4//LRP8// |
| GO:0034703 | cation channel complex | Cellular component | 14 | 217 | 395 | 18698 | 3.05398121682319 | 0.000222379466349736 | 0.0477559903986058 | 3.65290531629277 | 0.0354430379746835 | NLGN1//GRIN2B//GRIA3//SESTD1//KCNIP2//KCNA6//CACNG1//LRRC38//KCNIP3//ABCC8//CNTN2//KCNQ4//SCN3B//SCN1A// |
| GO:0098791 | Golgi subcompartment | Cellular component | 33 | 819 | 395 | 18698 | 1.9073399174665 | 0.000308874927375054 | 0.0530647125230343 | 3.51021734370437 | 0.0835443037974684 | YKT6//KDELR1//TMED2//VPS45//GBP4//AP1S1//DBNL//ST8SIA5//HLA-DPA1//GOLGA7B//NCAM1//NDFIP2//GALNT10//SLC30A6//RAB2A//XYLT2//ST3GAL3//SLC35A2//TNKS2//NDFIP1//FAM57B//CREB3L3//NDST2//NDST3//HS6ST1//GOSR2//TGOLN2//NUCB1//SYS1//ARFIP2//VPS53//CALN1//STEAP2// |
| GO:1902495 | transmembrane transporter complex | Cellular component | 17 | 321 | 395 | 18698 | 2.50692850664458 | 0.000510190074354415 | 0.072227919635107 | 3.2922679947561 | 0.0430379746835443 | GABRR1//BEST1//NLGN1//GRIN2B//GRIA3//SESTD1//KCNIP2//KCNA6//SCN3B//SCN1A//CACNG1//LRRC38//KCNIP3//ABCC8//CNTN2//KCNQ4//ATP1B4// |
| GO:0034702 | ion channel complex | Cellular component | 16 | 296 | 395 | 18698 | 2.55874101950051 | 0.000593679130985929 | 0.072227919635107 | 3.2264482171291 | 0.0405063291139241 | GABRR1//BEST1//NLGN1//GRIN2B//GRIA3//SESTD1//KCNIP2//KCNA6//SCN3B//SCN1A//CACNG1//LRRC38//KCNIP3//ABCC8//CNTN2//KCNQ4// |
| GO:1990351 | transporter complex | Cellular component | 17 | 329 | 395 | 18698 | 2.44596975876265 | 0.000672669798697155 | 0.072227919635107 | 3.17219807068737 | 0.0430379746835443 | NLGN1//GRIN2B//GRIA3//SESTD1//KCNIP2//KCNA6//GABRR1//BEST1//ATP1B4//SCN3B//SCN1A//CACNG1//LRRC38//KCNIP3//ABCC8//CNTN2//KCNQ4// |
| GO:0044424 | intracellular part | Cellular component | 329 | 14395 | 395 | 18698 | 1.08188796215282 | 0.00117620796711295 | 0.0915952747389474 | 2.92951588320513 | 0.832911392405063 | UBE4B//UBE2E1//GNAO1//GNAZ//GNA14//UBQLN1//PSMC6//ACTR2//MAEA//EMC8//PPIH//IFI44//CAMKK2//YKT6//ARPP19//ZNF268//TOMM34//PRDM4//NUDT4//KLHL2//SLAIN1//MSI2//LYPD6//CPS1//SRXN1//CTH//METTL21A//PPP4R2//PRUNE2//DAPK3//DLX5//EDN1//TET3//DNAH12//EVC//EWSR1//SKA3//FGFR2//AVL9//MGRN1//TTC28//LARP1//HAAO//CARHSP1//ARFIP2//FZD2//TXNDC8//SERBP1//MYCBP//HEYL//RND1//BMP10//RBMS3//DBNL//KCNIP2//CD209//EHD3//HMGB3//TLX2//IARS//ARHGAP40//KIF2A//KIT//MARK1//MEA1//TRIM67//MTAP//NFKB1//FOXP3//UFM1//PFDN1//PIK3C2A//RHOF//SGTB//NDFIP2//RALGPS2//PPP3CB//PRKAA1//PARVA//KMT2E//MAPK4//TULP4//TBC1D24//DDX55//PURA//PEX5//HIVEP3//RIT2//TRIM21//TTK//YWHAB//DDX39B//TNFAIP8L2//FZD3//TNKS2//SNN//CASP7//RNF135//RGS5//CYP4F2//BHLHE40//TNFSF14//FBP2//EMILIN3//ZNF804A//NREP//SH3BP5//EEF1E1//CDC5L//OXSR1//SLC23A2//SATB2//ALAD//BAG5//XRCC4//C2ORF49//HOXC9//KLHL14//ATP1B4//TIMM23//LETM2//GPD2//NDUFA12//POLR2E//OGT//MGST1//ERH//TMED2//AP1S1//CTNS//CYP7A1//HLA-DPA1//NOVA1//PALM//PAX5//FAR2//GRIPAP1//NUP214//SEPT7//PYURF//AKAP10//SLC9B1//ELK1//DDAH1//PHYH//LYRM2//CREBZF//CEP85//ABCC8//ARPC3//SGK2//VAV3//TGOLN2//CPLX1//RALBP1//TAGAP//SDR9C7//UBXN2B//DPYS//EIF4A2//EIF4G2//MAPRE1//PAN3//SULT4A1//STEAP2//GAS2//PTPN18//WDR91//KCNIP3//KRT9//NCAM1//NDN//SPRN//C9ORF78//ATP6V1G2//VPS53//MAP7D1//IL26//BRK1//WDFY1//TP53INP2//PCTP//RAB2A//CPLX3//BCR//SFRP1//SPTBN2//TLN1//UBE2D3//BEST1//VSNL1//CA7//ZNF322//PIP4K2C//CCDC6//CDK2AP1//KRTAP1-1//MEX3B//ACBD6//PARD6B//CREB3L3//KRT38//SYS1//ZW10//PAXBP1//ARHGEF6//AKAP7//VPS26A//GOSR2//CANX//EXTL2//ZDHHC20//LRRTM1//ITGA5//MEST//ASPH//DNAJB12//LRRC59//RTN4//THBS1//SLC35A2//MOGAT2//EDEM3//TMX1//FAM57B//KDELR1//NUCB1//VPS45//NLGN1//IGFBP1//GALNT10//SLC30A6//SLC1A6//NDFIP1//JAM3//CXCL14//SLC39A2//ANP32E//POLI//GBP4//TAF8//FAM168B//REP15//MLPH//CALN1//IGFBP7//GPX8//ANO8//FBXO45//ZSWIM6//SMPD1//SNAPC1//ASCL1//NFYA//WDR3//RPP14//ZNF618//LSM11//PASD1//RNF38//ZNF519//ZNF781//FOXG1//OTP//ZNF281//LDOC1//GABPA//FAM32A//PROX2//H3F3B//MCIDAS//C9ORF129//NAB1//PLAG1//RAG1//PRDM12//GPBP1L1//ZNF17//PAX8//IFRD2//AKIRIN1//BSN//ZNF468//ZNF682//MICAL2//ANKRD50//NPTX1//SSPN//EXD1//FERMT3//PTBP1//PPP1R26//SESTD1//ASF1B//GATAD2B//SNRNP27//LIN9//THUMPD1//SCN1A//SNRPA//MYOM3//LRP8//CGNL1//ST8SIA5//GOLGA7B//XYLT2//ST3GAL3//NDST2//NDST3//HS6ST1//SLC27A4//CYP4F3//INSIG2//EMC10//SYT9//PTAFR//SYNPR//SYPL2//GRIA3//GRP//SCN3B//LGALS3BP//VLDLR//DPEP3//TSPAN14//FAM206A//SCNM1//P2RX2//TOMM40L// |
| GO:0005737 | cytoplasm | Cellular component | 273 | 11544 | 395 | 18698 | 1.11944919603147 | 0.00120933152821803 | 0.0915952747389474 | 2.91745462458315 | 0.691139240506329 | FGFR2//ARFIP2//RND1//DBNL//RHOF//NDFIP1//PARD6B//CANX//THBS1//EXD1//CARHSP1//SEPT7//BSN//YKT6//KDELR1//TMED2//VPS45//GBP4//AP1S1//ST8SIA5//HLA-DPA1//GOLGA7B//NCAM1//NDFIP2//GALNT10//SLC30A6//RAB2A//XYLT2//ST3GAL3//SLC35A2//TNKS2//FAM57B//CREB3L3//NDST2//NDST3//HS6ST1//GOSR2//CEP85//PYURF//PAN3//TRIM21//MEX3B//VPS53//TXNDC8//KIT//DPEP3//CTNS//SMPD1//VPS26A//TIMM23//EMC8//TOMM34//AKAP10//SLC9B1//ELK1//DDAH1//MYCBP//MGST1//NFKB1//PHYH//NDUFA12//LYRM2//CREBZF//ABCC8//YWHAB//OGT//SH3BP5//BAG5//ARPC3//ACTR2//SGK2//VAV3//TGOLN2//CAMKK2//ZNF268//CPLX1//RALBP1//NUDT4//KLHL2//TAGAP//SDR9C7//UBXN2B//SRXN1//CTH//DPYS//EIF4A2//EIF4G2//ALAD//MAPRE1//MGRN1//HAAO//SULT4A1//SERBP1//STEAP2//GAS2//PTPN18//GNAZ//WDR91//UBQLN1//KCNIP3//EHD3//IARS//ARHGAP40//KIF2A//KRT9//MTAP//NDN//SPRN//PAX5//C9ORF78//PIK3C2A//ATP6V1G2//POLR2E//PPP3CB//PRKAA1//MAP7D1//PARVA//IL26//BRK1//MAPK4//GRIPAP1//TULP4//PSMC6//KLHL14//WDFY1//DDX55//PEX5//TP53INP2//PCTP//CPLX3//BCR//SFRP1//SPTBN2//TLN1//UBE2D3//UBE2E1//BEST1//VSNL1//XRCC4//CA7//ZNF322//PIP4K2C//NUP214//CCDC6//CDK2AP1//KRTAP1-1//CASP7//RNF135//ACBD6//RGS5//KRT38//FBP2//SYS1//ZW10//PAXBP1//ARHGEF6//AKAP7//EEF1E1//OXSR1//FAR2//SNN//TOMM40L//CPS1//LETM2//GPD2//VLDLR//ANKRD50//NUCB1//EXTL2//ZDHHC20//LRRTM1//ITGA5//MEST//ASPH//UFM1//DNAJB12//LRRC59//RTN4//MOGAT2//EDEM3//TMX1//IGFBP1//IGFBP7//GPX8//ANO8//SLC27A4//CYP7A1//CYP4F3//INSIG2//CYP4F2//NLGN1//SLC1A6//JAM3//CXCL14//EDN1//NPTX1//SSPN//SYT9//PTAFR//MAEA//MYOM3//REP15//LARP1//BMP10//SCN3B//SCN1A//SYNPR//SYPL2//GRIA3//FZD2//MLPH//LGALS3BP//FERMT3//SLC39A2//PALM//ANP32E//CALN1//ERH//GRP//TSPAN14//POLI//TAF8//FAM168B//CDC5L//AVL9//EMC10//UBE4B//PPIH//IFI44//ARPP19//PRDM4//SLAIN1//MSI2//LYPD6//METTL21A//PPP4R2//PRUNE2//DAPK3//DLX5//TET3//DNAH12//EVC//EWSR1//SKA3//TTC28//HEYL//RBMS3//KCNIP2//CD209//HMGB3//TLX2//MARK1//MEA1//TRIM67//FOXP3//PFDN1//SGTB//RALGPS2//KMT2E//TBC1D24//PURA//HIVEP3//RIT2//TTK//DDX39B//TNFAIP8L2//FZD3//BHLHE40//TNFSF14//EMILIN3//ZNF804A//NREP//SLC23A2// |
| GO:0098793 | presynapse | Cellular component | 18 | 379 | 395 | 18698 | 2.24818142346615 | 0.00124315220701777 | 0.0915952747389474 | 2.90547569467205 | 0.0455696202531646 | FBXO45//CPLX1//AP1S1//TBC1D24//CPLX3//CHRM1//ELK1//KCNIP3//KCNA6//FZD3//BSN//SYT9//ABCC8//SYNPR//SYPL2//ATP6V1G2//NLGN1//ZNF804A// |
| GO:0005622 | intracellular | Cellular component | 334 | 14673 | 395 | 18698 | 1.0775206678589 | 0.00130758874018426 | 0.0915952747389474 | 2.88352882784151 | 0.845569620253165 | SATB2//OGT//YKT6//KDELR1//TMED2//VPS45//GBP4//AP1S1//DBNL//ST8SIA5//HLA-DPA1//GOLGA7B//NCAM1//NDFIP2//GALNT10//SLC30A6//RAB2A//XYLT2//ST3GAL3//SLC35A2//TNKS2//NDFIP1//FAM57B//CREB3L3//NDST2//NDST3//HS6ST1//GOSR2//UBE4B//UBE2E1//CARHSP1//H3F3B//CEP85//GNAO1//GNAZ//GNA14//DDX39B//POLR2E//UBQLN1//PSMC6//PYURF//SKA3//TTK//ZW10//SEPT7//PURA//KMT2E//DLX5//GABPA//ASF1B//GATAD2B//ANP32E//TTC28//KIF2A//PAN3//TRIM21//MEX3B//VPS53//PAX5//TXNDC8//KIT//DPEP3//KRT9//KRT38//TET3//FERMT3//CTNS//SMPD1//VPS26A//TIMM23//EMC8//TOMM34//AKAP10//SLC9B1//ELK1//DDAH1//MYCBP//MGST1//NFKB1//PHYH//NDUFA12//LYRM2//CREBZF//ABCC8//YWHAB//SH3BP5//BAG5//ARPC3//ACTR2//SGK2//VAV3//TGOLN2//CAMKK2//ZNF268//CPLX1//RALBP1//NUDT4//KLHL2//TAGAP//SDR9C7//UBXN2B//SRXN1//CTH//DPYS//EIF4A2//EIF4G2//ALAD//MAPRE1//MGRN1//HAAO//ARFIP2//SULT4A1//SERBP1//STEAP2//GAS2//PTPN18//RND1//WDR91//KCNIP3//EHD3//IARS//ARHGAP40//MTAP//NDN//SPRN//C9ORF78//PIK3C2A//ATP6V1G2//RHOF//PPP3CB//PRKAA1//MAP7D1//PARVA//IL26//BRK1//MAPK4//GRIPAP1//TULP4//KLHL14//WDFY1//DDX55//PEX5//TP53INP2//PCTP//CPLX3//BCR//SFRP1//SPTBN2//TLN1//UBE2D3//BEST1//VSNL1//XRCC4//CA7//ZNF322//PIP4K2C//NUP214//CCDC6//CDK2AP1//KRTAP1-1//CASP7//RNF135//ACBD6//PARD6B//RGS5//FBP2//SYS1//PAXBP1//ARHGEF6//AKAP7//EEF1E1//OXSR1//FAR2//CANX//MAEA//WDR3//RPP14//ZNF618//TAF8//LSM11//PASD1//PPP4R2//RNF38//DAPK3//ZNF519//ZNF781//EWSR1//FGFR2//FOXG1//ATP1B4//OTP//ZNF281//LDOC1//FAM32A//HEYL//PROX2//HMGB3//TLX2//MCIDAS//ASCL1//C9ORF129//NAB1//NFYA//NOVA1//NUCB1//PALM//FOXP3//UFM1//PLAG1//RAG1//HIVEP3//PRDM12//RIT2//GPBP1L1//SNAPC1//ZNF17//PAX8//IFRD2//AKIRIN1//BHLHE40//BSN//ZNF468//ZNF682//ZNF804A//NREP//MICAL2//CDC5L//LRRC59//RTN4//P2RX2//PPIH//IFI44//ARPP19//PRDM4//SLAIN1//MSI2//LYPD6//CPS1//METTL21A//PRUNE2//EDN1//DNAH12//EVC//AVL9//LARP1//FZD2//BMP10//RBMS3//KCNIP2//CD209//MARK1//MEA1//TRIM67//PFDN1//SGTB//RALGPS2//TBC1D24//TNFAIP8L2//FZD3//SNN//CYP4F2//TNFSF14//EMILIN3//SLC23A2//SNRNP27//POLI//LIN9//HOXC9//THUMPD1//PTBP1//SCN1A//SNRPA//C2ORF49//PPP1R26//TOMM40L//LETM2//GPD2//VLDLR//ANKRD50//EXTL2//ZDHHC20//LRRTM1//ITGA5//MEST//ASPH//DNAJB12//THBS1//MOGAT2//EDEM3//TMX1//IGFBP1//IGFBP7//GPX8//ANO8//SLC27A4//CYP7A1//CYP4F3//INSIG2//NLGN1//SLC1A6//JAM3//CXCL14//NPTX1//SSPN//SYT9//PTAFR//MYOM3//LRP8//FBXO45//REP15//CGNL1//FAM206A//SCNM1//SCN3B//SYNPR//SYPL2//GRIA3//MLPH//LGALS3BP//SLC39A2//ZSWIM6//CALN1//ERH//GRP//TSPAN14//EXD1//SESTD1//FAM168B//EMC10//GPR3//GRIN2B//IL5RA//MAS1//CLEC1A// |
| GO:0097458 | neuron part | Cellular component | 50 | 1533 | 395 | 18698 | 1.5439239680613 | 0.00139346645189332 | 0.0915952747389474 | 2.85590348258971 | 0.126582278481013 | ACTR2//CHRM1//FBXO45//NLGN1//DBNL//GRIN2B//PALM//RTN4//BCR//LRP8//BSN//LYPD6//SYNPR//GABRR1//HTR3E//NPTX1//KLHL14//RIT2//SYT9//ABCC8//UNC5A//YKT6//CPLX1//ELK1//ASCL1//PURA//SCN1A//SPTBN2//CNTN2//FZD3//CANX//ZNF804A//NDN//CPLX3//GRIA3//FAM168B//LRRTM1//KCNA6//GRIPAP1//GNAO1//GNAZ//KCNIP3//FAM206A//MLPH//NDFIP1//AP1S1//TBC1D24//ARPC3//SYPL2//ATP6V1G2// |
| GO:0031984 | organelle subcompartment | Cellular component | 53 | 1655 | 395 | 18698 | 1.51591877318444 | 0.00149282170703756 | 0.0915952747389474 | 2.82599205849971 | 0.134177215189873 | TGOLN2//NUCB1//SYS1//RTN4//ASPH//AP1S1//ARFIP2//HLA-DPA1//VPS53//CALN1//TMED2//ST3GAL3//YKT6//KDELR1//VPS45//GBP4//DBNL//ST8SIA5//GOLGA7B//NCAM1//NDFIP2//GALNT10//SLC30A6//RAB2A//XYLT2//SLC35A2//TNKS2//NDFIP1//FAM57B//CREB3L3//NDST2//NDST3//HS6ST1//GOSR2//PYURF//SLC27A4//CYP7A1//EXTL2//CYP4F3//MEST//MGST1//INSIG2//LRRC59//KLHL14//MOGAT2//TMX1//CANX//CYP4F2//ZW10//STEAP2//DNAJB12//EMC8//EMC10// |
| GO:0005938 | cell cortex | Cellular component | 15 | 296 | 395 | 18698 | 2.39881970578173 | 0.00165759158322822 | 0.0949247446662027 | 2.78052246715133 | 0.0379746835443038 | BSN//MAEA//SEPT7//SPTBN2//ACTR2//MLPH//MAPRE1//ASPH//FGFR2//ARFIP2//RND1//DBNL//RHOF//NDFIP1//PARD6B// |
| GO:0036477 | somatodendritic compartment | Cellular component | 29 | 766 | 395 | 18698 | 1.79212083154311 | 0.00183798231146423 | 0.0986766753467359 | 2.73565867253122 | 0.0734177215189873 | YKT6//CPLX1//ELK1//ASCL1//RTN4//KLHL14//PURA//SCN1A//SPTBN2//CNTN2//LRP8//FZD3//CANX//ZNF804A//RIT2//UNC5A//NDN//CHRM1//NLGN1//GNAO1//GNAZ//DBNL//KCNIP3//FAM206A//GRIPAP1//MLPH//NDFIP1//BSN//PALM// |
| GO:0005794 | Golgi apparatus | Cellular component | 48 | 1501 | 395 | 18698 | 1.51376550653994 | 0.00258365828674223 | 0.123015691158499 | 2.58776492643382 | 0.121518987341772 | YKT6//KDELR1//TMED2//VPS45//GBP4//AP1S1//DBNL//ST8SIA5//HLA-DPA1//GOLGA7B//NCAM1//NDFIP2//GALNT10//SLC30A6//RAB2A//XYLT2//ST3GAL3//SLC35A2//TNKS2//NDFIP1//FAM57B//CREB3L3//NDST2//NDST3//HS6ST1//GOSR2//VPS53//NUCB1//TGOLN2//SYS1//STEAP2//ARFIP2//CALN1//UBXN2B//FGFR2//NLGN1//MAPRE1//ZDHHC20//TXNDC8//KCNIP3//IGFBP1//ITGA5//PIK3C2A//PEX5//CEP85//SLC1A6//JAM3//CXCL14// |
| GO:0044306 | neuron projection terminus | Cellular component | 9 | 139 | 395 | 18698 | 3.0649667607686 | 0.00281271478509052 | 0.123015691158499 | 2.55087430393196 | 0.0227848101265823 | CHRM1//ELK1//KCNIP3//KCNA6//CPLX1//AP1S1//TBC1D24//CPLX3//BSN// |
| GO:0044431 | Golgi apparatus part | Cellular component | 33 | 935 | 395 | 18698 | 1.67070737155622 | 0.00281418422931281 | 0.123015691158499 | 2.55064747507647 | 0.0835443037974684 | YKT6//KDELR1//TMED2//VPS45//GBP4//AP1S1//DBNL//ST8SIA5//HLA-DPA1//GOLGA7B//NCAM1//NDFIP2//GALNT10//SLC30A6//RAB2A//XYLT2//ST3GAL3//SLC35A2//TNKS2//NDFIP1//FAM57B//CREB3L3//NDST2//NDST3//HS6ST1//GOSR2//VPS53//NUCB1//ARFIP2//CALN1//SYS1//STEAP2//TGOLN2// |
| GO:0098796 | membrane protein complex | Cellular component | 39 | 1166 | 395 | 18698 | 1.58330329808715 | 0.00307576416740867 | 0.123015691158499 | 2.51204696689385 | 0.0987341772151899 | PYURF//PIK3C2A//TOMM40L//VPS26A//NDUFA12//YKT6//CPLX1//CPLX3//GOSR2//INSIG2//EMC8//EMC10//EVC//GNAO1//GNAZ//GNA14//SCN3B//SCN1A//ATP1B4//CACNG1//LRRC38//KCNIP3//KCNIP2//KCNA6//ABCC8//CNTN2//KCNQ4//SSPN//ITGA5//TIMM23//ATP6V1G2//AP1S1//NLGN1//GRIN2B//GRIA3//SESTD1//GABRR1//BEST1//HLA-DPA1// |
| GO:0014069 | postsynaptic density | Cellular component | 12 | 227 | 395 | 18698 | 2.50238108515028 | 0.00333626195684752 | 0.123015691158499 | 2.47673985673127 | 0.030379746835443 | GRIA3//GRIN2B//ACTR2//CHRM1//FBXO45//NLGN1//DBNL//PALM//RTN4//BCR//LRP8//BSN// |
| GO:0008076 | voltage-gated potassium channel complex | Cellular component | 7 | 92 | 395 | 18698 | 3.60170610897083 | 0.00334694059348216 | 0.123015691158499 | 2.47535199608317 | 0.0177215189873418 | LRRC38//KCNIP3//KCNIP2//KCNA6//ABCC8//CNTN2//KCNQ4// |
| GO:0098794 | postsynapse | Cellular component | 19 | 450 | 395 | 18698 | 1.99866104078762 | 0.0034749166050889 | 0.123015691158499 | 2.4590556136305 | 0.0481012658227848 | NLGN1//CANX//ZNF804A//CHRM1//FBXO45//GABRR1//GRIA3//GRIN2B//LRRTM1//BCR//SSPN//PALM//GRIPAP1//ACTR2//DBNL//RTN4//LRP8//BSN//P2RX2// |
| GO:0060076 | excitatory synapse | Cellular component | 4 | 30 | 395 | 18698 | 6.3115611814346 | 0.00348074435207641 | 0.123015691158499 | 2.45832787288338 | 0.010126582278481 | NLGN1//FGFR2//LRRTM1//BSN// |
| GO:0099572 | postsynaptic specialization | Cellular component | 12 | 229 | 395 | 18698 | 2.48052622851141 | 0.00358020055758147 | 0.123015691158499 | 2.4460926441351 | 0.030379746835443 | ACTR2//CHRM1//FBXO45//NLGN1//DBNL//GRIN2B//PALM//RTN4//BCR//LRP8//BSN//GRIA3// |
| GO:0042995 | cell projection | Cellular component | 61 | 2055 | 395 | 18698 | 1.40512858418799 | 0.00387200914296631 | 0.126396982305395 | 2.41206362586387 | 0.154430379746835 | KLHL2//ARFIP2//DBNL//ITGA5//TLN1//UBXN10//DNAH12//EVC//TULP4//LYPD6//SYNPR//GABRR1//HTR3E//GRIN2B//NPTX1//KLHL14//RIT2//ARPC3//ACTR2//BRK1//ARHGEF6//SEPT7//FAM168B//LRRTM1//KCNA6//PALM//GRIPAP1//LRP8//FZD3//CANX//BSN//CPLX1//CHRM1//ELK1//NLGN1//GNAO1//GNAZ//KCNIP3//FAM206A//PURA//MLPH//NDFIP1//ZNF804A//YKT6//SLC27A4//RNF38//TXNDC8//UNC5A//SLC9B1//KIF2A//EHD3//MAPRE1//SCN1A//CNTN2//AP1S1//TBC1D24//CPLX3//RTN4//CAMKK2//NDN//FERMT3// |
| GO:0032279 | asymmetric synapse | Cellular component | 12 | 232 | 395 | 18698 | 2.44845045831515 | 0.00397289699912185 | 0.126396982305395 | 2.40089269426829 | 0.030379746835443 | ACTR2//CHRM1//FBXO45//NLGN1//DBNL//GRIN2B//PALM//RTN4//BCR//LRP8//BSN//GRIA3// |
| GO:0098984 | neuron to neuron synapse | Cellular component | 12 | 235 | 395 | 18698 | 2.41719364395368 | 0.00439950944927966 | 0.134970664890401 | 2.35659574518377 | 0.030379746835443 | NLGN1//ACTR2//CHRM1//FBXO45//DBNL//GRIN2B//PALM//RTN4//BCR//LRP8//BSN//GRIA3// |
| GO:0043679 | axon terminus | Cellular component | 8 | 123 | 395 | 18698 | 3.07881033240712 | 0.00462040287534569 | 0.136859519652481 | 2.33532015454967 | 0.020253164556962 | CPLX1//AP1S1//TBC1D24//CPLX3//CHRM1//ELK1//KCNIP3//KCNA6// |
| GO:0034705 | potassium channel complex | Cellular component | 7 | 99 | 395 | 18698 | 3.34704002045774 | 0.00501942841435862 | 0.140171878703705 | 2.29934573517039 | 0.0177215189873418 | LRRC38//KCNIP3//KCNIP2//KCNA6//ABCC8//CNTN2//KCNQ4// |
| GO:0097447 | dendritic tree | Cellular component | 22 | 570 | 395 | 18698 | 1.82703086831002 | 0.00505858933622218 | 0.140171878703705 | 2.29597057582997 | 0.0556962025316456 | CPLX1//CHRM1//ELK1//NLGN1//GNAO1//GNAZ//DBNL//KCNIP3//FAM206A//GRIPAP1//PURA//LRP8//MLPH//FZD3//NDFIP1//BSN//YKT6//CANX//ZNF804A//RTN4//PALM//RIT2// |
| GO:0030424 | axon | Cellular component | 21 | 538 | 395 | 18698 | 1.84771540162816 | 0.00537948705254953 | 0.144405605566876 | 2.26925913341684 | 0.0531645569620253 | LRRTM1//ZNF804A//SCN1A//CNTN2//CPLX1//AP1S1//TBC1D24//CPLX3//CHRM1//ELK1//KCNIP3//KCNA6//RTN4//ARPC3//FAM168B//PALM//GRIPAP1//LRP8//FZD3//CANX//BSN// |
| GO:0030285 | integral component of synaptic vesicle membrane | Cellular component | 3 | 18 | 395 | 18698 | 7.88945147679325 | 0.00603055277366703 | 0.156977116138787 | 2.21964287765247 | 0.00759493670886076 | SYNPR//SYPL2//ATP6V1G2// |
| GO:0120025 | plasma membrane bounded cell projection | Cellular component | 58 | 1980 | 395 | 18698 | 1.38663086561821 | 0.00640521138508406 | 0.157699510771717 | 2.19346653307609 | 0.146835443037975 | KLHL2//ARFIP2//DBNL//ITGA5//TLN1//UBXN10//DNAH12//EVC//TULP4//LYPD6//SYNPR//GABRR1//HTR3E//GRIN2B//NPTX1//KLHL14//RIT2//ARPC3//ACTR2//BRK1//ARHGEF6//SEPT7//FAM168B//LRRTM1//KCNA6//PALM//GRIPAP1//LRP8//FZD3//CANX//BSN//CPLX1//CHRM1//ELK1//NLGN1//GNAO1//GNAZ//KCNIP3//FAM206A//PURA//MLPH//NDFIP1//SLC27A4//ZNF804A//YKT6//RNF38//TXNDC8//UNC5A//SLC9B1//KIF2A//EHD3//MAPRE1//SCN1A//CNTN2//AP1S1//TBC1D24//CPLX3//RTN4// |
| GO:0016020 | membrane | Cellular component | 224 | 9426 | 395 | 18698 | 1.12491224112138 | 0.00652486703507887 | 0.157699510771717 | 2.18542833403105 | 0.567088607594937 | VAV3//TGOLN2//SLC26A1//SLC27A4//PTPRT//AKAP10//CHRM1//PIK3IP1//FAM168B//LYPD6//SYT9//CTNS//SLC9B1//EFNA3//EWSR1//FGFR2//NLGN1//P2RX2//MGRN1//ARFIP2//FZD2//ZDHHC20//GABRR1//SERBP1//STEAP2//RND1//GNAO1//GNAZ//HTR3E//FFAR3//GRIA3//DBNL//GRIN2B//UBQLN1//SLC39A2//KCNIP3//KCNIP2//CD209//HLA-DPA1//CD300E//IL4R//IL5RA//ITGA5//KCNA6//KIT//MARK1//MAS1//MGST1//ASPH//NCAM1//NPTX1//SPRN//PALM//PIK3C2A//RHOF//RALGPS2//PPP3CB//PARVA//SCN3B//KMT2E//CYSLTR2//RTN4//PTAFR//TBC1D24//SEMA4G//ANO8//CPLX3//RIT2//SCN1A//ANO3//DPEP3//SFRP1//SLC1A6//SLC5A3//SMPD1//ABCC8//CNTN2//TECTB//TMPRSS2//UBE2D3//VLDLR//BEST1//LRP8//CACNG1//FZD3//TSPAN14//CALN1//JAM3//PARD6B//OGT//RGS5//TNFSF14//FBP2//UNC5A//KCNQ4//ZNF804A//GNA14//CD81//SLC23A2//PYURF//KDELR1//TMED2//CYP7A1//EXTL2//CYP4F3//MEST//INSIG2//LRRC59//KLHL14//RAB2A//XYLT2//MOGAT2//TMX1//CANX//FAM57B//CREB3L3//CYP4F2//ZW10//GOSR2//SSPN//WDR3//DNAJB12//AKIRIN1//PRKAA1//SPTBN2//SEPT7//MAPRE1//VPS45//EHD3//REP15//VPS53//NDFIP1//VPS26A//AP1S1//WDR91//GRIPAP1//NDFIP2//YKT6//YWHAB//ATP1B4//TIMM23//CPS1//LETM2//GPD2//NDUFA12//TOMM34//SNN//GBP4//ST8SIA5//GOLGA7B//GALNT10//SLC30A6//ST3GAL3//SLC35A2//TNKS2//NDST2//NDST3//HS6ST1//EVC//TLN1//PEX5//FBXO45//LRRTM1//BCR//NUCB1//SYS1//DAPK3//ATP6V1G2//SMIM12//TM4SF18//TMEM132E//TEDDM1//CCDC167//AVL9//CLEC9A//C16ORF54//EMC10//SMCO3//GPX8//MANSC1//TMEM248//LRRN1//SLC35E2B//TMEM246//PPP1R3F//MAEA//LRRC38//GPR3//GPR34//TRABD2B//CLEC1A//XG//TSPAN18//TOMM40L//FAR2//THBS1//SYNPR//SYPL2//CPLX1//SESTD1//EMC8//ARPC3//ACTR2//RALBP1//BROX//EIF4G2//LARP1//GAS2//IARS//KIF2A//KRT9//LGALS3BP//PSMC6//PTBP1//DDX55//RASL11B//TTK//VSNL1//RERGL//PIP4K2C//EDEM3//FERMT3//BAG5//CDC5L// |
| GO:0012505 | endomembrane system | Cellular component | 115 | 4421 | 395 | 18698 | 1.23133262134977 | 0.00660905982279606 | 0.157699510771717 | 2.17986031703672 | 0.291139240506329 | ATP1B4//GNAZ//LRRC59//RTN4//TNKS2//TGOLN2//YKT6//STEAP2//ANKRD50//SMPD1//VPS26A//TMED2//UBXN2B//EXTL2//ZDHHC20//UBQLN1//KCNIP3//LRRTM1//ITGA5//MEST//MGST1//ASPH//UFM1//NDFIP2//DNAJB12//KLHL14//THBS1//SLC35A2//MOGAT2//EDEM3//TMX1//CANX//FAM57B//CREB3L3//ZW10//VPS45//AP1S1//FGFR2//NLGN1//MAPRE1//TXNDC8//IGFBP1//PIK3C2A//VPS53//GALNT10//SLC30A6//PEX5//RAB2A//CEP85//SLC1A6//NDFIP1//JAM3//CXCL14//GOSR2//KDELR1//EDN1//NPTX1//SSPN//VLDLR//MGRN1//DBNL//NUCB1//WDFY1//CTNS//AVL9//REP15//GRIPAP1//KIT//DPEP3//SYT9//WDR3//AKIRIN1//WDR91//HLA-DPA1//RHOF//PTAFR//ALAD//GRP//NFKB1//ABCC8//FERMT3//PYURF//SLC27A4//CYP7A1//CYP4F3//INSIG2//XYLT2//CYP4F2//TSPAN14//ACTR2//LGALS3BP//EHD3//UBE2D3//IGFBP7//GPX8//ANO8//EMC8//EMC10//GBP4//ST8SIA5//GOLGA7B//NCAM1//ST3GAL3//NDST2//NDST3//HS6ST1//SYS1//ARFIP2//CALN1//NUP214//P2RX2//SYNPR//SYPL2//ATP6V1G2//RABL3// |
| GO:0043005 | neuron projection | Cellular component | 39 | 1224 | 395 | 18698 | 1.50827748821047 | 0.00685268396906312 | 0.159093392687168 | 2.16413929644129 | 0.0987341772151899 | FAM168B//LRRTM1//KCNA6//PALM//GRIPAP1//LRP8//FZD3//CANX//BSN//CPLX1//CHRM1//ELK1//NLGN1//GNAO1//GNAZ//DBNL//KCNIP3//FAM206A//PURA//MLPH//NDFIP1//UNC5A//RIT2//YKT6//ZNF804A//SCN1A//CNTN2//AP1S1//TBC1D24//CPLX3//RTN4//ARPC3//LYPD6//SYNPR//GABRR1//HTR3E//GRIN2B//NPTX1//KLHL14// |
| GO:0044425 | membrane part | Cellular component | 167 | 6780 | 395 | 18698 | 1.16596318285352 | 0.00731795875854192 | 0.162542987135466 | 2.13561004237444 | 0.422784810126582 | PYURF//KDELR1//TMED2//SLC27A4//CYP7A1//EXTL2//CYP4F3//MEST//MGST1//ASPH//INSIG2//LRRC59//RTN4//KLHL14//RAB2A//XYLT2//MOGAT2//TMX1//CANX//FAM57B//CREB3L3//CYP4F2//ZW10//GOSR2//AP1S1//VLDLR//TGOLN2//SLC26A1//TOMM34//PTPRT//VPS45//SMIM12//PIK3IP1//TM4SF18//TMEM132E//TEDDM1//FAM168B//LETM2//SYT9//CTNS//SLC9B1//CCDC167//EVC//FGFR2//P2RX2//AVL9//FZD2//ZDHHC20//CLEC9A//C16ORF54//EMC10//HTR3E//ST8SIA5//CD209//CD300E//LRRTM1//IL5RA//KCNA6//KIT//SMCO3//NCAM1//GPX8//NDFIP2//MANSC1//TMEM248//GALNT10//SLC30A6//SCN3B//PTAFR//LRRN1//SEMA4G//ANO8//ANO3//SFRP1//ST3GAL3//SLC5A3//SLC35E2B//SLC35A2//BEST1//LRP8//FZD3//NDFIP1//SNN//CALN1//JAM3//TMEM246//NDST2//TNFSF14//PPP1R3F//UNC5A//KCNQ4//NDST3//SLC23A2//LYPD6//EFNA3//SPRN//DPEP3//CNTN2//TECTB//CD81//NLGN1//ITGA5//THBS1//PPP3CB//SCN1A//PIK3C2A//TOMM40L//VPS26A//NDUFA12//YKT6//CPLX1//CPLX3//EMC8//ATP6V1G2//MAEA//CHRM1//LRRC38//ATP1B4//GABRR1//STEAP2//GPR3//GPR34//FFAR3//GRIN2B//SLC39A2//HLA-DPA1//IL4R//TRABD2B//MAS1//PALM//CLEC1A//CYSLTR2//SLC1A6//ABCC8//TMPRSS2//XG//SSPN//TSPAN14//TSPAN18//HS6ST1//FAR2//SYS1//DNAJB12//SYNPR//SYPL2//WDR91//NUCB1//TLN1//TIMM23//GNAO1//GNAZ//GNA14//CACNG1//KCNIP3//KCNIP2//PRKAA1//SPTBN2//PARD6B//SEPT7//MAPRE1//DAPK3//RIT2//FBXO45//GRIA3//BCR//EHD3//SESTD1// |
| GO:0030133 | transport vesicle | Cellular component | 16 | 381 | 395 | 18698 | 1.98789328549121 | 0.00737971652885118 | 0.162542987135466 | 2.13196032006316 | 0.0405063291139241 | HLA-DPA1//TGOLN2//STEAP2//SYT9//ABCC8//TMED2//GOSR2//SYNPR//SYPL2//ATP6V1G2//AP1S1//YKT6//KDELR1//EDN1//NPTX1//SSPN// |
| GO:0098588 | bounding membrane of organelle | Cellular component | 59 | 2040 | 395 | 18698 | 1.36905187391412 | 0.00775891025429106 | 0.166622597710901 | 2.11019927149724 | 0.149367088607595 | YKT6//KDELR1//TMED2//VPS45//GBP4//AP1S1//DBNL//ST8SIA5//HLA-DPA1//GOLGA7B//NCAM1//NDFIP2//GALNT10//SLC30A6//RAB2A//XYLT2//ST3GAL3//SLC35A2//TNKS2//NDFIP1//FAM57B//CREB3L3//NDST2//NDST3//HS6ST1//GOSR2//CTNS//SYT9//RHOF//PTAFR//STEAP2//EHD3//REP15//VPS53//UBE2D3//VPS26A//GRIA3//ASPH//EVC//NUCB1//ARFIP2//CALN1//SYS1//MGST1//TSPAN14//WDR91//GRIPAP1//TGOLN2//FZD2//PEX5//TOMM34//SNN//VLDLR//ABCC8//ATP6V1G2//TOMM40L//FAR2//SYNPR//SYPL2// |
| GO:0032588 | trans-Golgi network membrane | Cellular component | 6 | 85 | 395 | 18698 | 3.34141474311243 | 0.00922258380343073 | 0.193224377735293 | 2.03514738975232 | 0.0151898734177215 | AP1S1//ARFIP2//HLA-DPA1//VPS53//CALN1//SYS1// |
| GO:0030425 | dendrite | Cellular component | 21 | 569 | 395 | 18698 | 1.74704900892083 | 0.00986366628791703 | 0.201735460507636 | 2.00596162941393 | 0.0531645569620253 | CANX//NLGN1//ZNF804A//YKT6//RTN4//PALM//CPLX1//CHRM1//ELK1//GNAO1//GNAZ//DBNL//KCNIP3//FAM206A//GRIPAP1//PURA//LRP8//MLPH//FZD3//NDFIP1//BSN// |
| GO:0030662 | coated vesicle membrane | Cellular component | 9 | 171 | 395 | 18698 | 2.49140572951366 | 0.0106522474818623 | 0.211050406181645 | 1.97255875222572 | 0.0227848101265823 | TMED2//HLA-DPA1//GOSR2//KDELR1//TGOLN2//SYT9//DBNL//FZD2//AP1S1// |
| GO:0098797 | plasma membrane protein complex | Cellular component | 21 | 574 | 395 | 18698 | 1.73183081197901 | 0.0108104981047641 | 0.211050406181645 | 1.96615429502294 | 0.0531645569620253 | GNAO1//GNAZ//GNA14//SCN3B//SCN1A//ATP1B4//CACNG1//LRRC38//KCNIP3//KCNIP2//KCNA6//ABCC8//CNTN2//KCNQ4//SSPN//ITGA5//HLA-DPA1//NLGN1//GRIN2B//GRIA3//EVC// |
| GO:0150034 | distal axon | Cellular component | 12 | 268 | 395 | 18698 | 2.11955412809371 | 0.0119485217319742 | 0.223833073329606 | 1.92268582219358 | 0.030379746835443 | LRRTM1//ZNF804A//CHRM1//ELK1//KCNIP3//KCNA6//RTN4//CPLX1//AP1S1//TBC1D24//CPLX3//ARPC3// |
| GO:0044444 | cytoplasmic part | Cellular component | 226 | 9626 | 395 | 18698 | 1.11137504701139 | 0.0119864043925051 | 0.223833073329606 | 1.92131107433928 | 0.572151898734177 | CARHSP1//SEPT7//TIMM23//PYURF//EMC8//YKT6//TOMM34//AKAP10//SLC9B1//ELK1//DDAH1//MYCBP//MGST1//NFKB1//PHYH//NDFIP2//NDUFA12//LYRM2//CREBZF//CEP85//ABCC8//YWHAB//OGT//SH3BP5//BAG5//ARPC3//ACTR2//SGK2//VAV3//TGOLN2//CAMKK2//ZNF268//CPLX1//RALBP1//NUDT4//KLHL2//TAGAP//AP1S1//SDR9C7//UBXN2B//SRXN1//CTH//DPYS//EIF4A2//EIF4G2//ALAD//MAPRE1//MGRN1//HAAO//ARFIP2//PAN3//SULT4A1//SERBP1//STEAP2//GAS2//PTPN18//RND1//GNAZ//DBNL//WDR91//UBQLN1//KCNIP3//EHD3//IARS//ARHGAP40//KIF2A//KRT9//MTAP//NCAM1//NDN//SPRN//PAX5//C9ORF78//PIK3C2A//ATP6V1G2//POLR2E//RHOF//VPS53//PPP3CB//PRKAA1//MAP7D1//PARVA//IL26//BRK1//MAPK4//GRIPAP1//TULP4//PSMC6//KLHL14//WDFY1//DDX55//PEX5//TP53INP2//PCTP//RAB2A//CPLX3//BCR//SFRP1//SPTBN2//TRIM21//TLN1//UBE2D3//UBE2E1//BEST1//VSNL1//XRCC4//CA7//ZNF322//PIP4K2C//NUP214//CCDC6//TNKS2//CDK2AP1//KRTAP1-1//CASP7//MEX3B//RNF135//ACBD6//PARD6B//CREB3L3//RGS5//KRT38//FBP2//SYS1//ZW10//PAXBP1//ARHGEF6//AKAP7//EEF1E1//VPS26A//GOSR2//OXSR1//CANX//TMED2//EXTL2//ZDHHC20//LRRTM1//ITGA5//MEST//ASPH//UFM1//DNAJB12//LRRC59//RTN4//THBS1//SLC35A2//MOGAT2//EDEM3//TMX1//FAM57B//KDELR1//NUCB1//VPS45//FGFR2//NLGN1//TXNDC8//IGFBP1//GALNT10//SLC30A6//SLC1A6//NDFIP1//JAM3//CXCL14//GPD2//SLC39A2//PALM//ANP32E//ERH//POLI//GBP4//TAF8//FAM168B//REP15//MLPH//CALN1//CDC5L//ANKRD50//SMPD1//EDN1//NPTX1//SSPN//LARP1//EXD1//FAR2//CPS1//ST8SIA5//HLA-DPA1//GOLGA7B//XYLT2//ST3GAL3//NDST2//NDST3//HS6ST1//IGFBP7//GPX8//ANO8//SLC27A4//CYP7A1//CYP4F3//INSIG2//CYP4F2//EMC10//CTNS//MAEA//MYOM3//BMP10//SCN3B//SCN1A//KIT//AVL9//VLDLR//SYT9//PTAFR//GRIA3//FZD2//TSPAN14//BSN//SNN//LETM2//SYNPR//SYPL2//GRP//TOMM40L//LGALS3BP//FERMT3//DPEP3// |
| GO:0031224 | intrinsic component of membrane | Cellular component | 137 | 5506 | 395 | 18698 | 1.17782947946314 | 0.0130319168831548 | 0.232327777335522 | 1.8849916985969 | 0.346835443037975 | TGOLN2//SLC26A1//KDELR1//TOMM34//TMED2//SLC27A4//PTPRT//VPS45//SMIM12//PIK3IP1//TM4SF18//TMEM132E//TEDDM1//FAM168B//LETM2//SYT9//CTNS//SLC9B1//CCDC167//EVC//EXTL2//FGFR2//P2RX2//AVL9//FZD2//ZDHHC20//CLEC9A//C16ORF54//EMC10//HTR3E//ST8SIA5//CD209//CD300E//LRRTM1//IL5RA//KCNA6//KIT//CYP4F3//MEST//MGST1//SMCO3//ASPH//NCAM1//GPX8//NDFIP2//MANSC1//TMEM248//LRRC59//GALNT10//SLC30A6//SCN3B//PTAFR//LRRN1//SEMA4G//ANO8//ANO3//XYLT2//SFRP1//ST3GAL3//SLC5A3//SLC35E2B//SLC35A2//VLDLR//BEST1//LRP8//FZD3//MOGAT2//NDFIP1//TMX1//SNN//CALN1//JAM3//FAM57B//TMEM246//CREB3L3//NDST2//TNFSF14//PPP1R3F//UNC5A//KCNQ4//NDST3//GOSR2//SLC23A2//LYPD6//EFNA3//SPRN//DPEP3//CNTN2//TECTB//MAEA//YKT6//CHRM1//LRRC38//NLGN1//ATP1B4//GABRR1//STEAP2//GPR3//GPR34//FFAR3//GRIN2B//SLC39A2//HLA-DPA1//IL4R//TRABD2B//MAS1//PALM//CLEC1A//CYSLTR2//SLC1A6//ABCC8//TMPRSS2//XG//SSPN//TSPAN14//TSPAN18//HS6ST1//CD81//DNAJB12//RTN4//FAR2//SYS1//SYNPR//SYPL2//ATP6V1G2//CANX//TIMM23//TOMM40L//SCN1A//ITGA5//KCNIP3//KCNIP2//INSIG2//EMC8//CACNG1//GRIA3//SESTD1// |
| GO:0031301 | integral component of organelle membrane | Cellular component | 9 | 177 | 395 | 18698 | 2.40695129800472 | 0.0131272565740634 | 0.232327777335522 | 1.88182602632761 | 0.0227848101265823 | P2RX2//FAR2//STEAP2//SYS1//SYNPR//SYPL2//ATP6V1G2//TIMM23//TRABD2B// |
| GO:0044297 | cell body | Cellular component | 19 | 513 | 395 | 18698 | 1.75321143928739 | 0.0132526904417236 | 0.232327777335522 | 1.87769594622657 | 0.0481012658227848 | YKT6//CPLX1//ELK1//ASCL1//RTN4//KLHL14//PURA//SCN1A//SPTBN2//CNTN2//LRP8//FZD3//CANX//ZNF804A//UNC5A//NDN//GNAO1//GNAZ//RIT2// |
| GO:0030136 | clathrin-coated vesicle | Cellular component | 9 | 179 | 395 | 18698 | 2.38005798741249 | 0.0140402215721497 | 0.239450873778174 | 1.85262603844534 | 0.0227848101265823 | TGOLN2//STEAP2//SYT9//DBNL//EDN1//FZD2//HLA-DPA1//AP1S1//PIK3C2A// |
| GO:0033116 | endoplasmic reticulum-Golgi intermediate compartment membrane | Cellular component | 5 | 68 | 395 | 18698 | 3.48064035740879 | 0.0144168897612732 | 0.239450873778174 | 1.84112842236344 | 0.0126582278481013 | YKT6//KDELR1//TMED2//RAB2A//GOSR2// |
| GO:0030135 | coated vesicle | Cellular component | 12 | 276 | 395 | 18698 | 2.05811777655476 | 0.0147726610927764 | 0.239450873778174 | 1.83054126543207 | 0.030379746835443 | TMED2//PIK3C2A//TGOLN2//STEAP2//EDN1//KDELR1//HLA-DPA1//GOSR2//SYT9//DBNL//FZD2//AP1S1// |
| GO:0030054 | cell junction | Cellular component | 38 | 1247 | 395 | 18698 | 1.44249794443373 | 0.0147740352854985 | 0.239450873778174 | 1.83050086806859 | 0.0962025316455696 | ITGA5//KIT//EIF4G2//JAM3//RND1//PARD6B//CGNL1//SCN1A//ARPC3//ACTR2//FZD2//EHD3//PARVA//TLN1//YWHAB//NUP214//CD81//CHRM1//LYPD6//SYNPR//SYT9//FBXO45//NLGN1//GABRR1//GRIA3//DBNL//GRIN2B//LRRTM1//GRIPAP1//TBC1D24//CPLX3//BCR//SPTBN2//NDFIP1//SSPN//FERMT3//FBP2//BSN// |
| GO:0031410 | cytoplasmic vesicle | Cellular component | 63 | 2279 | 395 | 18698 | 1.30856193866953 | 0.0151425769907739 | 0.240879141390274 | 1.81980020953633 | 0.159493670886076 | TGOLN2//YKT6//STEAP2//ANKRD50//SMPD1//VPS26A//NUCB1//KDELR1//TMED2//EDN1//NPTX1//SSPN//EHD3//MGRN1//DBNL//WDFY1//CTNS//AVL9//REP15//VPS53//PIK3C2A//ATP6V1G2//RAB2A//YWHAB//CANX//THBS1//GRIPAP1//TXNDC8//KIT//DPEP3//SYT9//WDR91//GOSR2//NDFIP2//HLA-DPA1//RHOF//PTAFR//ALAD//GRP//NFKB1//GRIA3//ABCC8//FERMT3//TSPAN14//MGST1//ACTR2//LGALS3BP//FZD2//AP1S1//VPS45//UBE2D3//NDFIP1//SYNPR//SYPL2//FGFR2//RND1//UBQLN1//SLC39A2//ITGA5//PALM//TP53INP2//TRIM21//ANP32E// |
| GO:0097708 | intracellular vesicle | Cellular component | 63 | 2282 | 395 | 18698 | 1.30684165566514 | 0.0155389924735084 | 0.242690809722613 | 1.80857714366772 | 0.159493670886076 | FGFR2//RND1//UBQLN1//SLC39A2//ITGA5//PALM//RHOF//TP53INP2//TRIM21//ANP32E//TGOLN2//YKT6//STEAP2//ANKRD50//SMPD1//VPS26A//NUCB1//KDELR1//TMED2//EDN1//NPTX1//SSPN//EHD3//MGRN1//DBNL//WDFY1//CTNS//AVL9//REP15//VPS53//GRIPAP1//PIK3C2A//THBS1//ATP6V1G2//RAB2A//YWHAB//CANX//TXNDC8//KIT//DPEP3//SYT9//WDR91//GOSR2//NDFIP2//HLA-DPA1//PTAFR//ALAD//GRP//NFKB1//GRIA3//ABCC8//FERMT3//TSPAN14//MGST1//ACTR2//LGALS3BP//FZD2//VPS45//UBE2D3//NDFIP1//AP1S1//SYNPR//SYPL2// |
| GO:0030018 | Z disc | Cellular component | 7 | 125 | 395 | 18698 | 2.65085569620253 | 0.0169509925368459 | 0.254494292599447 | 1.77080486733375 | 0.0177215189873418 | MYOM3//BMP10//PPP3CB//PARVA//SCN3B//SCN1A//FBP2// |
| GO:0016021 | integral component of membrane | Cellular component | 133 | 5369 | 395 | 18698 | 1.17261729902794 | 0.0170149536033137 | 0.254494292599447 | 1.76916923080868 | 0.336708860759494 | MAEA//YKT6//SLC26A1//CHRM1//LRRC38//EFNA3//FGFR2//NLGN1//P2RX2//ATP1B4//GABRR1//STEAP2//GPR3//HTR3E//GPR34//FFAR3//GRIN2B//SLC39A2//HLA-DPA1//IL4R//KCNA6//KIT//TRABD2B//MAS1//PALM//CLEC1A//CYSLTR2//PTAFR//SLC1A6//SLC5A3//ABCC8//CNTN2//TMPRSS2//XG//SSPN//TSPAN14//TSPAN18//HS6ST1//CD81//SLC23A2//TOMM40L//DNAJB12//RTN4//CANX//FAR2//SYS1//SYNPR//SYPL2//ATP6V1G2//TIMM23//SCN3B//SCN1A//ITGA5//KCNIP3//KCNIP2//KCNQ4//INSIG2//EMC8//EMC10//CACNG1//GRIA3//SESTD1//BEST1//TGOLN2//KDELR1//TOMM34//TMED2//SLC27A4//PTPRT//VPS45//SMIM12//PIK3IP1//TM4SF18//TMEM132E//TEDDM1//FAM168B//LETM2//SYT9//CTNS//SLC9B1//CCDC167//EVC//EXTL2//AVL9//FZD2//ZDHHC20//CLEC9A//C16ORF54//ST8SIA5//CD209//CD300E//LRRTM1//IL5RA//CYP4F3//MEST//MGST1//SMCO3//ASPH//NCAM1//GPX8//NDFIP2//MANSC1//TMEM248//LRRC59//GALNT10//SLC30A6//LRRN1//SEMA4G//ANO8//ANO3//XYLT2//SFRP1//ST3GAL3//SLC35E2B//SLC35A2//VLDLR//LRP8//FZD3//MOGAT2//NDFIP1//TMX1//SNN//CALN1//JAM3//FAM57B//TMEM246//CREB3L3//NDST2//TNFSF14//PPP1R3F//UNC5A//NDST3//GOSR2// |
| GO:0043226 | organelle | Cellular component | 306 | 13597 | 395 | 18698 | 1.06531094442836 | 0.0171835494421047 | 0.254494292599447 | 1.76488712316472 | 0.774683544303797 | UBXN10//DNAH12//EVC//TULP4//SEPT7//TMED2//AP1S1//CTNS//CYP7A1//FGFR2//RND1//HLA-DPA1//NOVA1//PALM//PAX5//RHOF//NDFIP2//FAR2//GRIPAP1//NUP214//CYP4F2//SPRN//PIK3C2A//VPS26A//CD81//ACTR2//CHRM1//FBXO45//NLGN1//DBNL//GRIN2B//RTN4//BCR//LRP8//BSN//TIMM23//PYURF//EMC8//YKT6//TOMM34//AKAP10//SLC9B1//ELK1//DDAH1//MYCBP//MGST1//NFKB1//PHYH//NDUFA12//LYRM2//CREBZF//CEP85//ABCC8//YWHAB//OGT//SH3BP5//BAG5//PEX5//UBE4B//MAEA//CAMKK2//ZNF268//WDR3//RPP14//NUDT4//ZNF618//GBP4//TAF8//LSM11//UBXN2B//PASD1//PPP4R2//RNF38//DAPK3//ZNF519//ZNF781//TET3//ALAD//EWSR1//FOXG1//MGRN1//SATB2//ATP1B4//OTP//ZNF281//LDOC1//GABPA//FAM32A//SERBP1//HEYL//PROX2//H3F3B//KCNIP3//EHD3//HMGB3//TLX2//MCIDAS//KIF2A//KRT9//ASCL1//C9ORF129//MTAP//NAB1//NFYA//NUCB1//FOXP3//UFM1//PLAG1//POLR2E//PRKAA1//KMT2E//MAPK4//PSMC6//WDFY1//DDX55//PURA//TP53INP2//RAB2A//RAG1//HIVEP3//PRDM12//RIT2//GPBP1L1//SNAPC1//TRIM21//UBE2E1//SLC35A2//XRCC4//ZNF17//PAX8//IFRD2//DDX39B//AKIRIN1//ZNF322//TNKS2//CDK2AP1//ANP32E//CASP7//PARD6B//CREB3L3//BHLHE40//ZNF468//ZNF682//ZNF804A//ZW10//NREP//PAXBP1//AKAP7//EEF1E1//MICAL2//CDC5L//UBQLN1//PIP4K2C//EXTL2//ZDHHC20//GNAZ//LRRTM1//ITGA5//MEST//ASPH//DNAJB12//LRRC59//KLHL14//THBS1//MOGAT2//EDEM3//TMX1//CANX//FAM57B//KDELR1//TGOLN2//VPS45//MAPRE1//TXNDC8//IGFBP1//VPS53//GALNT10//SLC30A6//SLC1A6//NDFIP1//JAM3//CXCL14//GOSR2//SLC39A2//ARPC3//VAV3//PIK3IP1//FAM168B//CPNE8//BROX//CTH//DPYS//HAAO//CARHSP1//CD209//IARS//CD300E//IGFBP7//TRABD2B//LGALS3BP//NCAM1//BRK1//PTBP1//SFRP1//SMPD1//TLN1//TMPRSS2//C1QA//UBE2D3//MLPH//ABHD8//CRISPLD1//FERMT3//KRT38//FBP2//GNA14//OXSR1//SKA3//TTK//SDR9C7//CPS1//EIF4A2//PPP1R26//MAP7D1//SLAIN1//MARK1//TRIM67//PARVA//CCDC6//KLHL2//POLI//SESTD1//STEAP2//ANKRD50//EDN1//NPTX1//SSPN//KIT//AVL9//REP15//DPEP3//SYT9//ATP6V1G2//PAN3//MEX3B//LARP1//EXD1//VLDLR//WDR91//PTAFR//GRP//GRIA3//TTC28//MYOM3//GAS2//CGNL1//SPTBN2//TSPAN14//CYP4F3//FZD2//ST8SIA5//GOLGA7B//XYLT2//ST3GAL3//NDST2//NDST3//HS6ST1//DLX5//ASF1B//GATAD2B//P2RX2//SGK2//PPIH//ARPP19//SNRNP27//METTL21A//PTPN18//LIN9//HOXC9//NDN//C9ORF78//PPP3CB//THUMPD1//SCN1A//SNRPA//C2ORF49//SNN//TOMM40L//LETM2//GPD2//GPX8//ANO8//SLC27A4//INSIG2//SYS1//FAM206A//SCNM1//BMP10//SCN3B//SYNPR//SYPL2//ARFIP2//CALN1//PRDM4//KRTAP1-1//EMC10// |
| GO:0072546 | ER membrane protein complex | Cellular component | 2 | 10 | 395 | 18698 | 9.4673417721519 | 0.0179063797245549 | 0.260704748871062 | 1.7469922100514 | 0.00506329113924051 | EMC8//EMC10// |
| GO:0033267 | axon part | Cellular component | 14 | 353 | 395 | 18698 | 1.87737655538423 | 0.0184579916609926 | 0.262545431643643 | 1.73381555455593 | 0.0354430379746835 | LRRTM1//ZNF804A//SCN1A//CNTN2//CPLX1//AP1S1//TBC1D24//CPLX3//CHRM1//ELK1//KCNIP3//KCNA6//ARPC3//RTN4// |
| GO:0098563 | intrinsic component of synaptic vesicle membrane | Cellular component | 3 | 27 | 395 | 18698 | 5.25963431786217 | 0.0187971898716844 | 0.262545431643643 | 1.72590707171724 | 0.00759493670886076 | SYNPR//SYPL2//ATP6V1G2// |
| GO:0032991 | protein-containing complex | Cellular component | 126 | 5071 | 395 | 18698 | 1.17618326098515 | 0.0189497284771896 | 0.262545431643643 | 1.72239700847707 | 0.318987341772152 | DDX39B//SKA3//TTK//ZW10//CDC5L//DBNL//FERMT3//C1QA//THBS1//PURA//SATB2//ALAD//LRP8//ARPC3//ACTR2//EIF4A2//EIF4G2//PSMC6//PFDN1//CGNL1//LIN9//YWHAB//IARS//EEF1E1//UBXN10//NUP214//SEPT7//BRK1//PAN3//LARP1//XRCC4//KCNIP3//NFYA//NFKB1//WDR3//MAEA//FGFR2//P2RX2//IL4R//KIT//VLDLR//C2ORF49//KIF2A//VPS53//EXD1//DNAH12//H3F3B//SSPN//ITGA5//PYURF//PIK3C2A//TOMM40L//VPS26A//NDUFA12//YKT6//CPLX1//CPLX3//GOSR2//INSIG2//EMC8//EMC10//EVC//CANX//PPIH//SNRPA//LSM11//MSI2//GATAD2B//ATP6V1G2//SNAPC1//ASCL1//AP1S1//MYOM3//PASD1//RPP14//CARHSP1//TRIM21//MEX3B//POLI//GABRR1//TAF8//GNAO1//GNAZ//GNA14//SCN3B//SCN1A//TIMM23//ATP1B4//CACNG1//LRRC38//KCNIP2//KCNA6//ABCC8//CNTN2//KCNQ4//HLA-DPA1//UBE4B//UBE2E1//GOLGA7B//GPD2//BEST1//PPP3CB//PPP4R2//UBQLN1//ERH//PRDM4//FBXO45//ZSWIM6//KLHL2//KLHL14//NLGN1//GRIN2B//GRIA3//POLR2E//OGT//SESTD1//PRKAA1//ANP32E//AKAP10//METTL21A//NAT9//FOXP3//ASF1B//KMT2E//PEX5//AKAP7// |
| GO:0031982 | vesicle | Cellular component | 110 | 4372 | 395 | 18698 | 1.19099679201362 | 0.0212034939101849 | 0.283656351205382 | 1.67359257016137 | 0.278481012658228 | FGFR2//RND1//UBQLN1//SLC39A2//ITGA5//PALM//RHOF//TP53INP2//TRIM21//ANP32E//ARPC3//ACTR2//VAV3//YKT6//PIK3IP1//AP1S1//FAM168B//CPNE8//BROX//CTH//CTNS//DPYS//ALAD//EXTL2//MGRN1//HAAO//DDAH1//CARHSP1//TXNDC8//SERBP1//GNAZ//DBNL//H3F3B//CD209//IARS//CD300E//IGFBP7//KRT9//TRABD2B//LGALS3BP//MEST//MTAP//NCAM1//NUCB1//UFM1//PIK3C2A//BRK1//PSMC6//RTN4//PTBP1//RAB2A//BCR//SFRP1//SMPD1//THBS1//TLN1//TMPRSS2//C1QA//UBE2D3//YWHAB//MLPH//ABHD8//PIP4K2C//CANX//CRISPLD1//FERMT3//PARD6B//KRT38//FBP2//EEF1E1//VPS26A//GNA14//CD81//SEPT7//OXSR1//TGOLN2//STEAP2//ANKRD50//WDFY1//TMED2//KDELR1//EDN1//NPTX1//SSPN//EHD3//ATP6V1G2//AVL9//REP15//VPS53//GRIPAP1//KIT//DPEP3//SYT9//WDR91//GOSR2//NDFIP2//HLA-DPA1//PTAFR//GRP//NFKB1//GRIA3//ABCC8//TSPAN14//MGST1//FZD2//VPS45//NDFIP1//SYNPR//SYPL2//SPRN// |
| GO:0005885 | Arp2/3 protein complex | Cellular component | 2 | 11 | 395 | 18698 | 8.60667433831991 | 0.0215831838599462 | 0.283656351205382 | 1.66588448964322 | 0.00506329113924051 | ARPC3//ACTR2// |
| GO:0017146 | NMDA selective glutamate receptor complex | Cellular component | 2 | 11 | 395 | 18698 | 8.60667433831991 | 0.0215831838599462 | 0.283656351205382 | 1.66588448964322 | 0.00506329113924051 | NLGN1//GRIN2B// |
| GO:0031300 | intrinsic component of organelle membrane | Cellular component | 9 | 193 | 395 | 18698 | 2.20741129402505 | 0.0217943180204368 | 0.283656351205382 | 1.66165671621248 | 0.0227848101265823 | TRABD2B//STEAP2//SYS1//P2RX2//FAR2//SYNPR//SYPL2//ATP6V1G2//TIMM23// |
| GO:0030665 | clathrin-coated vesicle membrane | Cellular component | 6 | 104 | 395 | 18698 | 2.73096397273612 | 0.0230053414187025 | 0.294949078786051 | 1.63817131705434 | 0.0151898734177215 | FZD2//HLA-DPA1//AP1S1//TGOLN2//SYT9//DBNL// |
| GO:0031201 | SNARE complex | Cellular component | 4 | 52 | 395 | 18698 | 3.6412852969815 | 0.0239549056236896 | 0.29855401893698 | 1.62060553581362 | 0.010126582278481 | CPLX1//YKT6//CPLX3//GOSR2// |
| GO:0030863 | cortical cytoskeleton | Cellular component | 6 | 105 | 395 | 18698 | 2.7049547920434 | 0.0239816383080927 | 0.29855401893698 | 1.62012115135142 | 0.0151898734177215 | MLPH//MAPRE1//BSN//MAEA//SPTBN2//ACTR2// |
| GO:0044448 | cell cortex part | Cellular component | 8 | 166 | 395 | 18698 | 2.28128717401251 | 0.0249851689970877 | 0.306603716692833 | 1.6023177086887 | 0.020253164556962 | MAEA//SEPT7//SPTBN2//ACTR2//ASPH//MLPH//MAPRE1//BSN// |
| GO:0017101 | aminoacyl-tRNA synthetase multienzyme complex | Cellular component | 2 | 12 | 395 | 18698 | 7.88945147679325 | 0.025542610217207 | 0.309029608120857 | 1.59273472394787 | 0.00506329113924051 | IARS//EEF1E1// |
| GO:0031674 | I band | Cellular component | 7 | 138 | 395 | 18698 | 2.40113740598055 | 0.0273968987455605 | 0.32686022253384 | 1.56229859535178 | 0.0177215189873418 | MYOM3//BMP10//PPP3CB//PARVA//SCN3B//SCN1A//FBP2// |
| GO:0005887 | integral component of plasma membrane | Cellular component | 47 | 1678 | 395 | 18698 | 1.32587921123701 | 0.0281471269213231 | 0.331210712676939 | 1.55056592855314 | 0.118987341772152 | SCN3B//SCN1A//ITGA5//ATP1B4//LRRC38//KCNIP3//KCNIP2//KCNA6//ABCC8//CNTN2//KCNQ4//NLGN1//GRIN2B//GRIA3//MAEA//YKT6//SLC26A1//CHRM1//EFNA3//FGFR2//P2RX2//GABRR1//STEAP2//GPR3//HTR3E//GPR34//FFAR3//SLC39A2//HLA-DPA1//IL4R//KIT//TRABD2B//MAS1//PALM//CLEC1A//CYSLTR2//PTAFR//SLC1A6//SLC5A3//TMPRSS2//XG//SSPN//TSPAN14//TSPAN18//HS6ST1//CD81//SLC23A2// |
| GO:0030658 | transport vesicle membrane | Cellular component | 9 | 203 | 395 | 18698 | 2.09867182141298 | 0.0289521394184568 | 0.336079564330465 | 1.5383193385583 | 0.0227848101265823 | TMED2//HLA-DPA1//GOSR2//SYT9//ABCC8//SYNPR//SYPL2//ATP6V1G2//AP1S1// |
| GO:0030140 | trans-Golgi network transport vesicle | Cellular component | 3 | 32 | 395 | 18698 | 4.4378164556962 | 0.0295118046403889 | 0.337437956682029 | 1.5300042326756 | 0.00759493670886076 | AP1S1//TGOLN2//STEAP2// |
| GO:0043025 | neuronal cell body | Cellular component | 16 | 449 | 395 | 18698 | 1.68683149615179 | 0.0298548133967802 | 0.337437956682029 | 1.52498563896895 | 0.0405063291139241 | UNC5A//NDN//YKT6//CPLX1//ELK1//ASCL1//RTN4//KLHL14//PURA//SCN1A//SPTBN2//CNTN2//LRP8//FZD3//CANX//ZNF804A// |
| GO:0015629 | actin cytoskeleton | Cellular component | 17 | 487 | 395 | 18698 | 1.65241078158709 | 0.0304426524009704 | 0.339613485875761 | 1.51651751113532 | 0.0430379746835443 | DBNL//FERMT3//MYOM3//DAPK3//GAS2//ARPC3//ACTR2//CGNL1//MLPH//SEPT7//MAEA//SPTBN2//ZNF268//KLHL2//RND1//PARVA//KLHL14// |
| GO:0005798 | Golgi-associated vesicle | Cellular component | 8 | 174 | 395 | 18698 | 2.17640040739124 | 0.0318322911746569 | 0.350563309218337 | 1.49713210128789 | 0.020253164556962 | TMED2//TGOLN2//STEAP2//KDELR1//HLA-DPA1//GOSR2//AP1S1//NUCB1// |
| GO:0031226 | intrinsic component of plasma membrane | Cellular component | 48 | 1741 | 395 | 18698 | 1.30509019259992 | 0.0338897476108303 | 0.366173994979996 | 1.46993166549817 | 0.121518987341772 | MAEA//YKT6//SLC26A1//CHRM1//LRRC38//EFNA3//FGFR2//NLGN1//P2RX2//ATP1B4//GABRR1//STEAP2//GPR3//HTR3E//GPR34//FFAR3//GRIN2B//SLC39A2//HLA-DPA1//IL4R//KCNA6//KIT//TRABD2B//MAS1//PALM//CLEC1A//CYSLTR2//PTAFR//SLC1A6//SLC5A3//ABCC8//CNTN2//TMPRSS2//XG//SSPN//TSPAN14//TSPAN18//HS6ST1//CD81//SLC23A2//SCN3B//SCN1A//ITGA5//KCNIP3//KCNIP2//KCNQ4//GRIA3//UNC5A// |
| GO:0001518 | voltage-gated sodium channel complex | Cellular component | 2 | 14 | 395 | 18698 | 6.7623869801085 | 0.0342556979720732 | 0.366173994979996 | 1.46526717914936 | 0.00506329113924051 | SCN3B//SCN1A// |
| GO:0002102 | podosome | Cellular component | 3 | 34 | 395 | 18698 | 4.17676842889054 | 0.0345286304928751 | 0.366173994979996 | 1.4618206467065 | 0.00759493670886076 | ACTR2//DBNL//FERMT3// |
| GO:0036126 | sperm flagellum | Cellular component | 5 | 86 | 395 | 18698 | 2.75213423609067 | 0.0355541220594883 | 0.367963745169885 | 1.44911004095641 | 0.0126582278481013 | SEPT7//SLC9B1//KIF2A//RNF38//TXNDC8// |
| GO:0097729 | 9+2 motile cilium | Cellular component | 5 | 86 | 395 | 18698 | 2.75213423609067 | 0.0355541220594883 | 0.367963745169885 | 1.44911004095641 | 0.0126582278481013 | RNF38//TXNDC8//SEPT7//SLC9B1//KIF2A// |
| GO:0044463 | cell projection part | Cellular component | 39 | 1377 | 395 | 18698 | 1.34069110063153 | 0.0376322673749888 | 0.378795356661313 | 1.42443961429293 | 0.0987341772151899 | CPLX1//CHRM1//ELK1//NLGN1//GNAO1//GNAZ//DBNL//KCNIP3//FAM206A//GRIPAP1//PURA//LRP8//MLPH//FZD3//NDFIP1//BSN//MAPRE1//CANX//ZNF804A//YKT6//EHD3//SLC27A4//PALM//ITGA5//TLN1//UNC5A//EVC//LRRTM1//SCN1A//CNTN2//AP1S1//TBC1D24//CPLX3//KCNA6//RTN4//ARPC3//SEPT7//SLC9B1//KIF2A// |
| GO:0120038 | plasma membrane bounded cell projection part | Cellular component | 39 | 1377 | 395 | 18698 | 1.34069110063153 | 0.0376322673749888 | 0.378795356661313 | 1.42443961429293 | 0.0987341772151899 | CPLX1//CHRM1//ELK1//NLGN1//GNAO1//GNAZ//DBNL//KCNIP3//FAM206A//GRIPAP1//PURA//LRP8//MLPH//FZD3//NDFIP1//BSN//MAPRE1//CANX//ZNF804A//YKT6//SLC27A4//PALM//ITGA5//TLN1//UNC5A//EVC//LRRTM1//SCN1A//CNTN2//AP1S1//TBC1D24//CPLX3//KCNA6//ARPC3//RTN4//SEPT7//SLC9B1//KIF2A//EHD3// |
| GO:0030176 | integral component of endoplasmic reticulum membrane | Cellular component | 7 | 148 | 395 | 18698 | 2.23889839206295 | 0.0379236329137054 | 0.378795356661313 | 1.42109006588033 | 0.0177215189873418 | INSIG2//HLA-DPA1//CANX//EMC8//EMC10//DNAJB12//RTN4// |
| GO:0005662 | DNA replication factor A complex | Cellular component | 2 | 15 | 395 | 18698 | 6.3115611814346 | 0.0389836372680609 | 0.38053345924164 | 1.40911764259774 | 0.00506329113924051 | PURA//CDC5L// |
| GO:0033268 | node of Ranvier | Cellular component | 2 | 15 | 395 | 18698 | 6.3115611814346 | 0.0389836372680609 | 0.38053345924164 | 1.40911764259774 | 0.00506329113924051 | SCN1A//CNTN2// |
| GO:0005834 | heterotrimeric G-protein complex | Cellular component | 3 | 36 | 395 | 18698 | 3.94472573839662 | 0.0399568586106482 | 0.381366017183853 | 1.39840866313098 | 0.00759493670886076 | GNAO1//GNAZ//GNA14// |
| GO:1905360 | GTPase complex | Cellular component | 3 | 36 | 395 | 18698 | 3.94472573839662 | 0.0399568586106482 | 0.381366017183853 | 1.39840866313098 | 0.00759493670886076 | GNAO1//GNAZ//GNA14// |
| GO:0099568 | cytoplasmic region | Cellular component | 16 | 468 | 395 | 18698 | 1.61834902088067 | 0.041073453494311 | 0.387715346721024 | 1.38643877972567 | 0.0405063291139241 | FGFR2//ARFIP2//RND1//DBNL//RHOF//NDFIP1//PARD6B//CANX//SEPT7//BSN//MAEA//SPTBN2//ACTR2//MLPH//MAPRE1//ASPH// |
| GO:0031227 | intrinsic component of endoplasmic reticulum membrane | Cellular component | 7 | 152 | 395 | 18698 | 2.17998001332445 | 0.042787541922447 | 0.399505418601978 | 1.36868266234011 | 0.0177215189873418 | DNAJB12//RTN4//HLA-DPA1//CANX//INSIG2//EMC8//EMC10// |
| GO:0005793 | endoplasmic reticulum-Golgi intermediate compartment | Cellular component | 6 | 123 | 395 | 18698 | 2.30910774930534 | 0.0464684323048302 | 0.426212830332917 | 1.33284197897037 | 0.0151898734177215 | YKT6//KDELR1//TMED2//RAB2A//GOSR2//NUCB1// |
| GO:0031090 | organelle membrane | Cellular component | 75 | 2950 | 395 | 18698 | 1.20347564900236 | 0.0471239608407906 | 0.426212830332917 | 1.32675821357313 | 0.189873417721519 | WDR3//DNAJB12//AKIRIN1//YKT6//AP1S1//YWHAB//ATP1B4//TIMM23//CPS1//LETM2//GPD2//NDUFA12//TOMM34//MGST1//SNN//KDELR1//TMED2//VPS45//GBP4//DBNL//ST8SIA5//HLA-DPA1//GOLGA7B//NCAM1//NDFIP2//GALNT10//SLC30A6//RAB2A//XYLT2//ST3GAL3//SLC35A2//TNKS2//NDFIP1//FAM57B//CREB3L3//NDST2//NDST3//HS6ST1//GOSR2//CTNS//SYT9//RHOF//PTAFR//STEAP2//EHD3//REP15//VPS53//UBE2D3//VPS26A//GRIA3//ASPH//EVC//NUCB1//ARFIP2//CALN1//SYS1//VLDLR//ABCC8//TSPAN14//WDR91//GRIPAP1//TGOLN2//FZD2//PEX5//ATP6V1G2//P2RX2//FAR2//SYNPR//SYPL2//TRABD2B//TOMM40L//CYP7A1//CYP4F3//LRRC59//CYP4F2// |
| GO:0044432 | endoplasmic reticulum part | Cellular component | 37 | 1319 | 395 | 18698 | 1.32786825462328 | 0.0474656517648619 | 0.426212830332917 | 1.323620551324 | 0.0936708860759494 | PYURF//TGOLN2//IGFBP1//IGFBP7//NUCB1//GPX8//ANO8//THBS1//EDEM3//CANX//KDELR1//TMED2//SLC27A4//CYP7A1//EXTL2//CYP4F3//MEST//MGST1//ASPH//INSIG2//LRRC59//RTN4//KLHL14//RAB2A//XYLT2//MOGAT2//TMX1//FAM57B//CREB3L3//CYP4F2//ZW10//GOSR2//EMC8//EMC10//EDN1//DNAJB12//HLA-DPA1// |
| GO:0032433 | filopodium tip | Cellular component | 2 | 17 | 395 | 18698 | 5.56902457185406 | 0.0491211527391837 | 0.426212830332917 | 1.30873145004294 | 0.00506329113924051 | NLGN1//FZD3// |
| GO:0043186 | P granule | Cellular component | 2 | 17 | 395 | 18698 | 5.56902457185406 | 0.0491211527391837 | 0.426212830332917 | 1.30873145004294 | 0.00506329113924051 | EXD1//CARHSP1// |
| GO:0045495 | pole plasm | Cellular component | 2 | 17 | 395 | 18698 | 5.56902457185406 | 0.0491211527391837 | 0.426212830332917 | 1.30873145004294 | 0.00506329113924051 | EXD1//CARHSP1// |
| GO:0060293 | germ plasm | Cellular component | 2 | 17 | 395 | 18698 | 5.56902457185406 | 0.0491211527391837 | 0.426212830332917 | 1.30873145004294 | 0.00506329113924051 | EXD1//CARHSP1// |
